# The Out-of-Pocket Cost Burden of Cancer Care—A Systematic Literature Review

**DOI:** 10.3390/curroncol28020117

**Published:** 2021-03-15

**Authors:** Nicolas Iragorri, Claire de Oliveira, Natalie Fitzgerald, Beverley Essue

**Affiliations:** 1 Institute of Health Policy, Management & Evaluation, Dalla Lana School of Public Health, University of Toronto, Toronto, ON M5T 3M7, Canada; claire.deoliveira@partnershipagainstcancer.ca (C.d.O.); beverley.essue@utoronto.ca (B.E.); 2 The Canadian Partnership Against Cancer, Toronto, ON M5H 1J8, Canada; natalie.fitzgerald@partnershipagainstcancer.ca; 3 Centre for Health Economics and Hull York Medical School, University of York, Heslington, York YO10 5DD, UK; 4 Centre for Addiction and Mental Health, Institute for Mental Health Policy Research and Campbell Family Mental Health Research Institute, Toronto, ON M6J 1H4, Canada

**Keywords:** out-of-pocket costs, economic burden, cancer, financial hardship, catastrophic expenditure

## Abstract

Background: Out-of-pocket costs pose a substantial economic burden to cancer patients and their families. The purpose of this study was to evaluate the literature on out-of-pocket costs of cancer care. Methods: A systematic literature review was conducted to identify studies that estimated the out-of-pocket cost burden faced by cancer patients and their caregivers. The average monthly out-of-pocket costs per patient were reported/estimated and converted to 2018 USD. Costs were reported as medical and non-medical costs and were reported across countries or country income levels by cancer site, where possible, and category. The out-of-pocket burden was estimated as the average proportion of income spent as non-reimbursable costs. Results: Among all cancers, adult patients and caregivers in the U.S. spent between USD 180 and USD 2600 per month, compared to USD 15–400 in Canada, USD 4–609 in Western Europe, and USD 58–438 in Australia. Patients with breast or colorectal cancer spent around USD 200 per month, while pediatric cancer patients spent USD 800. Patients spent USD 288 per month on cancer medications in the U.S. and USD 40 in other high-income countries (HICs). The average costs for medical consultations and in-hospital care were estimated between USD 40–71 in HICs. Cancer patients and caregivers spent 42% and 16% of their annual income on out-of-pocket expenses in low- and middle-income countries and HICs, respectively. Conclusions: We found evidence that cancer is associated with high out-of-pocket costs. Healthcare systems have an opportunity to improve the coverage of medical and non-medical costs for cancer patients to help alleviate this burden and ensure equitable access to care.

## 1. Introduction

Cancer is a major international health issue due to its considerable impact on mortality and morbidity. Over 22 million people are expected to be diagnosed with cancer in 2030, worldwide [1]. Similar to other chronic conditions, cancer patients require long-term medical attention, posing a considerable economic burden to healthcare systems, patients and their families [2]. Furthermore, rising costs of cancer care have been associated with higher out-of-pocket expenses, medical debt, and even bankruptcy [3]. As such, there is an imperative to understand and measure the economic burden to help mitigate the impact of cancer [4].

Conceptually, the economic burden of cancer can be divided into three categories: psychosocial costs, indirect costs (mostly productivity losses), and direct costs [5]. In turn, direct costs can be divided into medical and non-medical costs paid either by third-party payers (e.g., healthcare systems or private insurers), or by patients out-of-pocket. Studies have extensively evaluated the direct medical costs associated with cancer that are paid by healthcare systems [6,7]. However, there are less data on the medical and non-medical out-of-pocket expenses borne by cancer patients and their caregivers across international settings. Studies that have measured the out-of-pocket burden of cancer have usually focused on estimating a given cost category (e.g., medication copayments) among specific cancer patients (e.g., breast cancer survivors) from a single country perspective [8]. However, cancer is a heterogeneous condition, and the out-of-pocket burden is expected to depend on multiple factors, such as cancer site, patient age and sex, or insurance coverage arrangements in place in each context. Previous research has shown that out-of-pocket costs are expected to pose a heavier burden among cancer populations with lower income [9]. Moreover, out-of-pocket costs contribute to the economic burden of cancer patients, regardless of the country they live in. Although healthcare insurance coverage differs across jurisdictions, the literature suggests that medical debt is not just a problem in low- and middle-income countries (LMICs); it also extends to insured individuals in high-income countries (HICs) [10]. This is specifically due to new and costly therapies that create a greater demand on strained resources [11]. A synthesis of the evidence presents the opportunity to characterize and compare the out-of-pocket burden across settings, to help identify at-risk populations and understand which specific types of out-of-pocket expenses contribute more/less to the burden. Therefore, the objective of this study was to provide a comprehensive overview of the international literature on out-of-pocket costs associated with cancer and to provide a source that compiles these data and discusses the associated strengths and weaknesses of measuring these costs across diverse patient populations.

## 2. Methods

### 2.1. Data Sources and Search Strategies

A systematic review of electronic databases was conducted to identify studies, which estimated costs paid out-of-pocket by patients with cancer and their caregivers. In particular, we searched MEDLINE, EMBASE (Excerpta Medical Database), EconLit, and CINAHL, between database inception and 7 May 2019. Search terms combined medical subject headings (MeSH), Embase subject headings (Emtree), and keywords for out-of-pocket costs (e.g., deductibles, copayments), and cancer. No electronic search filters for date or language were used. The reference lists of all included papers were reviewed to identify potentially relevant papers. Google Scholar was searched using keywords from the main search strategy. The search strategies can be found in Supplementary 1. The review was registered in Prospero (ID: CRD42019133508). We followed the Preferred Reporting Items for Systematic Reviews and Meta-Analyses (PRISMA) guidelines [12]; the checklist can be found in Supplementary 4.

### 2.2. Eligibility Criteria

We included any study that estimated out-of-pocket costs for patients with any type of cancer, paid either by patients or their caregiver(s). No restriction was applied to the study design or the type of cost (e.g., medication, transport, etc). Costs were identified across the entire cancer care continuum from diagnosis to end-of-life care. Studies were excluded if any of the following criterion was met: (a) the population of interest was not cancer patients or their caregivers; (b) out-of-pocket costs were not explicitly estimated as a primary or secondary outcome; (c) the studies included duplicate data sources; and (d) a full-text article was unavailable. The search results were screened first by title and abstract, then by full text by two independent reviewers (NI and BE). Any article that either reviewer included at the title and abstract review stage was included for full-text review. The kappa statistic was estimated to evaluate inter-observer agreement [13]. Disagreements between reviewers were settled by discussion with a third reviewer (CdO) until a consensus was reached. 

### 2.3. Data Extraction

A data extraction template was designed from a sample of studies that measured different dimensions of the economic burden of cancer. We extracted the following study characteristics: authors, publication year, setting, country, data sources, study population, sample size, cancer site, cancer care continuum stage, mean age of patients, percentage of female population, percentage insured, and mean income of patients. The outcomes of interest were non-reimbursed medical and non-medical out-of-pocket costs, however defined. This included non-reimbursed co-payments and deductibles. The tool (e.g., surveys, cost diaries), time frame, currency, and currency year were extracted to estimate mean monthly out-of-pocket costs. Authors were contacted if further information was required. 

### 2.4. Data Synthesis

The out-of-pocket costs reported by individual studies were reported and synthesized. Studies that estimated mean out-of-pocket costs per month per patient and reported the standard deviation were extracted and did not require further synthesis. Standard deviations were estimated from confidence intervals assuming critical values of t distributions [14]. Median estimates were transformed to mean costs using mathematical inequalities and statistical approximations, as described by Hozo et al. [15]. To do so, studies had to report a median cost, the interquartile range (or range), and the sample size. To ensure comparability, all mean costs were transformed to reflect monthly expenditure (e.g., annual mean out-of-pocket costs were divided by 12 to obtain a mean per-month estimate). Furthermore, exchange rates were used to convert all non-USD costs to USD costs, which were then adjusted for inflation to establish a single metric to allow controlling for any changes in nominal prices. Exchange rates and pharma consumer price indices from the World Bank’s Global Economic Monitor were used to convert costs to 2018 USD [16]. Once all costs were converted to a single measure (mean out-of-pocket cost per month per patient), estimates were stratified and presented separately by country, country income-level (as defined by the World Bank [17]), or type of healthcare system (e.g., HICs with and without universal health coverage), depending on data availability; where possible, estimates were stratified and presented by cancer site within country. Costs were reported or estimated only from studies that provided sufficient information (i.e., currency, currency year, mean cost, standard deviation/measure of spread, time frame). Studies that failed to provide a measure of spread (e.g., standard deviation), or a time frame, could not be used to compute a weighted average. Furthermore, we estimated a weighted average cost across expenditure categories (medications, medical consultations, in-hospital care, transport/travel, and caregiver costs) and across cancer sites. Finally, the proportion of household income spent on out-of-pocket expenses for cancer-related care was reported and calculated for the studies that reported a measure of income (distribution or mean value) among the studied population

### 2.5. Quality Assessment

Quality assessment was conducted in duplicate (NI and BE) using the Ottawa-Newcastle Assessment Tool for Cohort Studies [18]. Cross-sectional studies were evaluated with a variation of the Ottawa-Newcastle tool [18]. Three domains were evaluated for prospective cohort and cross-sectional studies: selection (i.e., representativeness of the sample), comparability (i.e., comparability of subjects, confounding factors), and outcome (i.e., assessment of outcome, statistical test used). Each domain was assessed for risk of bias (low, unclear, or high) by two reviewers (NI and BE).

## 3. Results

**Figure 1:**
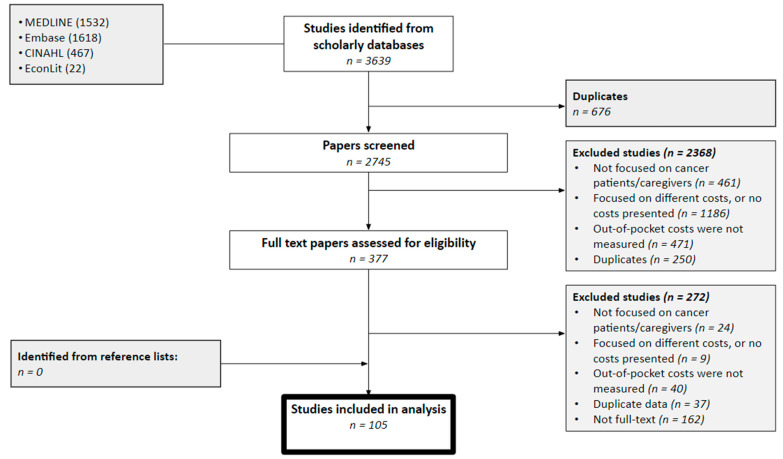
Preferred Reported Items for Systematic Reviews and Meta-Analyses (PRISMA) diagram. Note: This diagram shows the flow of information through the different sections of the systematic review, including the identified, excluded and included studies after the title/abstract and full-text reviews.

The systematic review identified 3639 records, of which 105 full-text studies were retrieved [8,19,20,21,22,23,24,25,26,27,28,29,30,31,32,33,34,35,36,37,38,39,40,41,42,43,44,45,46,47,48,49,50,51,52,53,54,55,56,57,58,59,60,61,62,63,64,65,66,67,68,69,70,71,72,73,74,75,76,77,78,79,80,81,82,83,84,85,86,87,88,89,90,91,92,93,94,95,96,97,98,99,100,101,102,103,104,105,106,107,108,109,110,111,112,113,114,115,116,117,118,119,120,121,122]. The eligibility criteria and reasons for exclusion are presented in Figure 1. Duplicate records (*n* = 676) were excluded before the abstract review stage. Half of the reviewed abstracts reported costs that were not relevant (e.g., indirect costs) and 20% did not measure out-of-pocket costs. In total, 377 studies were selected for full-text review, of which 42% were not full-text articles (i.e., conference abstracts). No additional records were identified after searching the reference lists of the included articles. A high inter-observer agreement was measured for the title and abstract review and the full-text review (kappa = 0.71).

The study characteristics are summarized in Table 1. The year of publication ranged from 1979 to 2019. The total combined sample size of the identified studies was 774,135 cancer patients and/or caregivers and ranged from 11 to 200,000. The studies with the largest sample size usually identified patients through administrative data sources, such as linked cancer registries, medical claims data, and medical expenditure surveys. Costs were collected retrospectively in most studies (*n* = 73) using observational and cross-sectional study designs. On the other hand, prospective studies followed cohorts of cancer patients through time (*n* = 32). The mean age of pediatric cancer patients ranged from 5.6 to 9 years old, and from 37 to 80 years old among adults. Half of the studies were conducted in the U.S. (*n* = 55, 52%), followed by Australia (*n* = 12, 11%), Western Europe (France, Germany, Ireland, UK, and Italy) (*n* = 11, 10%), Canada (*n* = 9, 8%), and India (*n* = 6, 5%). A few were conducted in South East Asia (Laos, Vietnam, Malaysia, Philippines, Thailand, Cambodia and Myanmar) (*n* = 4, 4%), China (*n* = 3, 3%), Japan (*n* = 3, 3%) and in Latin America (Mexico) (*n* = 1, 1%). Half of the studies (*n* = 54) included patients with full or partial healthcare insurance (public healthcare systems with universal coverage, private, or a combination) and excluded uninsured patients. All patients from studies conducted in countries with universal healthcare coverage were publicly insured. 

Most studies included patients receiving active treatment (any stage) (*n* = 50, 47%), followed by those on patients who were recently diagnosed (*n* = 25, 24%). A few studies focused on end-of-life and/or palliative care (*n* = 9, 8%), and survivorship (*n* = 9, 8%). Out-of-pocket costs were measured using different tools; some studies, usually those following cohorts of cancer patients, employed cost diaries and logbooks that patients used to register the out-of-pocket and non-reimbursed expenses related to their cancer care [20,23,59,68,75,78]. On the other hand, most observational studies were conducted using health administrative data, expenditure surveys, and medical expenditure claims from insurance companies and healthcare records.

**Figure 2:**
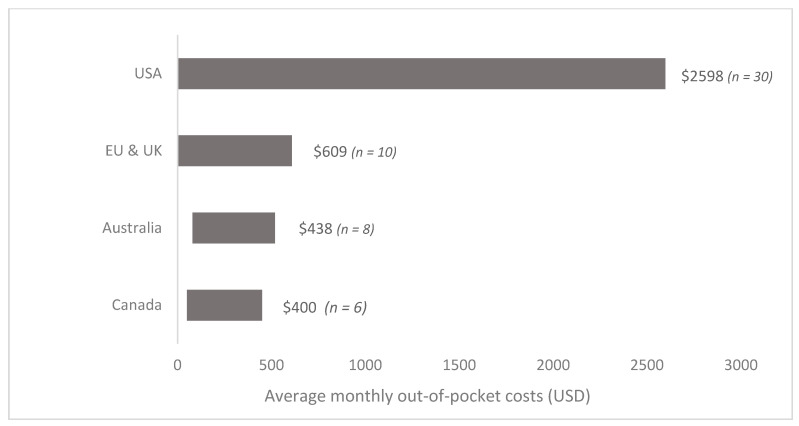
Range of monthly out-of-pocket costs per patient by country. Countries in the European Union (EU) include Italy, France, Ireland, and Germany. Costs are expressed in 2018 USD. Not enough data were available to include a range of costs (in 2018 USD) for other countries. Average costs per patient per month were also estimated: USD 300 in the U.S., USD 200 in Canada, USD 180 in the E.U. and USD 70 in Australia Not enough data were available to stratify these estimates by cancer site.

**Figure 3:**
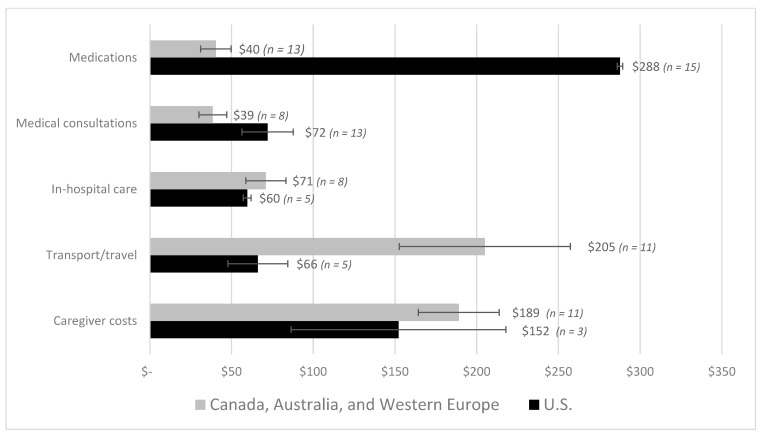
Average monthly out-of-pocket costs per patient by spending categories. Note: The medical expenditure categories were defined as prescription or over-the-counter drugs and medications, home and clinical medical visits, and in-hospital care. The non-medical categories included transport, travel and lodging, and formal and informal caregiver costs (e.g., daycare for pediatric patients). Costs are presented for comparison between the U.S. and countries with universal healthcare coverage. Not enough data was available to estimate costs for low- and middle-income countries. Costs are expressed in 2018 USD. Not enough data were available to stratify these estimates by cancer site.

Table 2 and Supplementary 2 summarize the individual out-of-pocket estimates across the 105 identified studies. Sixty-four estimates were reported and converted to mean out-of-pocket monthly costs per patient (2018 USD) for comparison through stratified analyses. Figure 2 summarizes the range of out-of-pocket costs estimated across countries for all cancer populations. Estimates for all cancers were lumped by country as there were not enough studies to present the findings by cancer site. The out-of-pocket cost for all adult cancer patients in the U.S ranged from USD 180 to USD 2598 per patient per month (around USD 300 per patient per month on average). Estimates for Western Europe (Germany, France, Ireland, Italy, and the UK) ranged between USD 4 and USD 609 per patient per month (average of USD 200 per patent per month). In Canada, costs ranged between USD 15 to USD 400 per patient per month (average of USD 187 per patient per month). Finally, the average out-of-pocket cost in Australia ranged between USD 58 and USD 438 per patient per month (average of USD 70). There was not enough information to estimate a range of costs (measured in 2018 USD) among studies conducted in other HICs (e.g., Japan), or in LMICs (Mexico, India, China, Vietnam, Thailand, etc.). Figure 3 summarizes the mean out-of-pocket cost for different expenditure categories among HICs (U.S., Germany, France, Italy, UK, Ireland, Canada and Australia). Furthermore, given the small number of studies by country, estimates were stratified by type of health-care system; that is, costs were reported separately for the U.S. and countries with universal healthcare coverage (Australia, Canada, and Western Europe) (unfortunately, estimates could not be presented by cancer site within country). In terms of non-reimbursable medical costs, the category that represented the highest out-of-pocket burden for the U.S. was medications, with an average monthly out-of-pocket cost per person of USD 288 (*n* = 15), compared with USD 40 (*n* = 13) in Canada, Australia, and Western Europe (combined). This was followed by expenditures in medical consultations (USD 72, *n* = 13), which was almost twice as high relative to countries with universal healthcare coverage (USD 39, *n* = 8). Finally, spending related to in-hospital care was similar between the two groups (~USD 60 and USD 70). Results were also estimated for non-medical expenditure categories. The out-of-pocket costs spent on travel/transportation and supportive care provided by caregivers were higher in countries with universal healthcare coverage compared with the U.S. (USD 205 vs. USD 66 and USD 189 vs. USD 152, respectively). Individual cost estimates per category were summarized and are presented in Supplementary 3.

Although most studies estimated costs across different categories, some focused on specific types of out-of-pocket costs. Several studies estimated medication costs only and exclusively followed patients throughout the cancer treatment pathway. These studies estimated the deductibles or co-payments associated with specific cancer medications (e.g., imatinib, bevacizumab) [86,100]. On the other hand, other studies focused on travel costs for outpatient treatment, which included non-medical fees associated with parking, lodging, accommodation, and public transportation [33,42,67,77,106]. Finally, a few studies identified other types of out-of-pocket costs such as medical devices, food, hair accessories, laboratory tests, and clothing [19,89,93,115]. However, insufficient data was provided to estimate a weighted mean for these categories.

**Figure 4:**
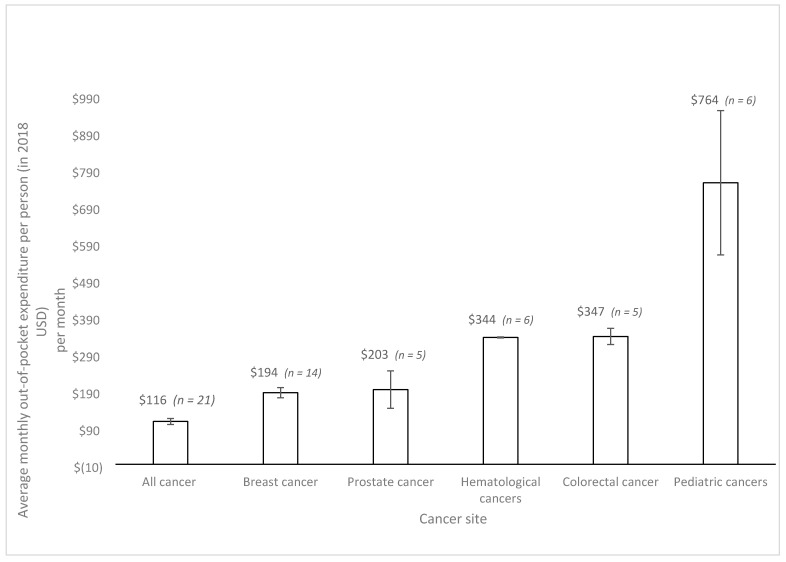
Average monthly out-of-pocket costs per patient by cancer site with 95% confidence intervals from high-income countries. Note: Studies that included patients with multiple cancer sites are reported under the ‘All cancer’ and ‘pediatric cancer’ categories. Costs are expressed in 2018 USD. Not enough data were available to report average costs per cancer site for low- and middle-income countries, or for individual high-income countries.

**Figure 5:**
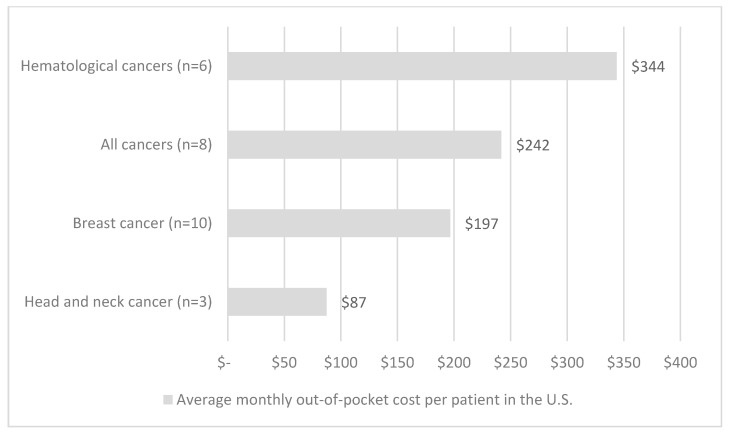
Average monthly out-of-pocket costs per patient by cancer site in the U.S. Note: Studies that included patients with multiple cancer sites are reported under the ‘All cancer’ category. Costs are expressed in 2018 USD. Not enough data were available to report average costs per cancer site for low- and middle-income countries, or for other high-income countries.

The distribution of the identified patient populations across cancer sites was as follows: most studies (*n* = 33, 31%) evaluated all adult, followed by breast (*n* = 18, 17%), leukemia (*n* = 11, 10%), all pediatric (*n* = 8, 7%), colorectal (*n* = 6, 5%), lung (*n* = 5, 5%), head and neck (*n* = 4, 4%), prostate (*n* = 4, 4%), ovarian (*n* = 3, 3%), pancreatic (*n* = 2, 2%), anal (*n* = 1, 1%), and brain cancers (*n* = 1, 1%). Figure 4 and Figure 5 summarize the estimated costs across cancer sites.). Mean weighted costs were estimated and combined for all HICs (U.S., Canada, Australia, Italy, France, Germany, UK, Japan) (Figure 4) and estimated for the U.S. (Figure 5) across cancer sites due to lack of data; moreover, there was not enough data from LMICs. Breast and prostate cancer patients faced similar out-of-pocket costs at around USD 200 per patient per month. On the other hand, the mean costs were slightly higher for hematological and colorectal cancers, estimated at around USD 400 per month per patient. The highest average out-of-pocket cost was estimated among pediatric populations and their caregivers, at an estimated USD 800 per month. This represents a four-fold difference compared with breast and prostate cancers, and a two-fold difference compared with colorectal and hematological cancers. 

**Figure 6:**
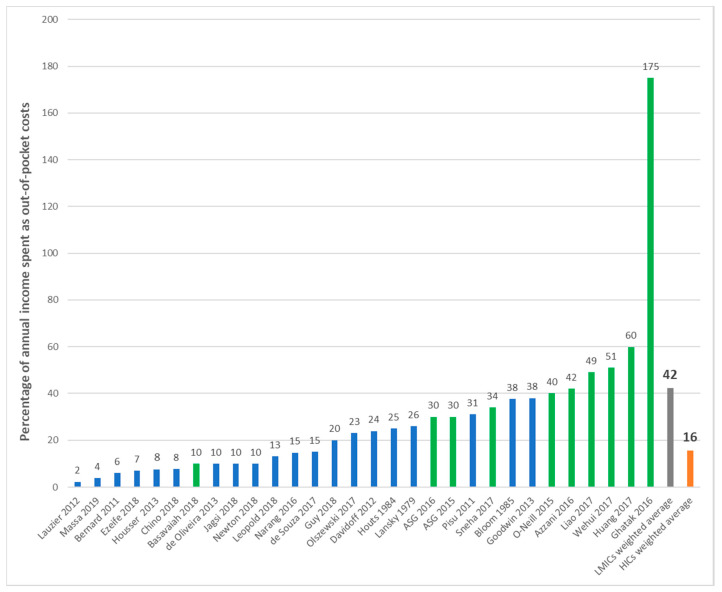
Average out-of-pocket costs per patient as a percentage of income. Legend: LMIC = low- and middle-income countries; HIC = high-income countries; ASG = Action Study Group; blue bars represent studies from HICs; green bars represent studies from LMICs. Note: This figure shows the costs from individual studies that estimated out-of-pocket expenditures relative to annual income. A weighted average was calculated for high-income countries (in green) and low-and middle- income countries (blue). Studies conducted in high-income countries include the U.S., Canada, and Australia. Studies conducted in low- and middle-income countries include China, Malaysia, India, Haiti, Brunei, Thailand, Indonesia, Philippines, Vietnam, Laos, Cambodia, and Myanmar.

We reported and estimated the total out-of-pocket costs as a proportion of the annual income in 33 studies (Table 2). Figure 6 summarizes these estimates per study and country income-level and presents a weighted average for HICs (U.S., Canada, Australia) and LMICs (China, Malaysia, India, Haiti, Brunei, Thailand, Indonesia, Philippines, Vietnam, Laos, Cambodia, Myanmar). Cancer patients and caregivers in HICs spent, on average, 16% of their annual income on out-of-pocket expenses related to cancer care, compared with 42% among LMICs. Most studies conducted in LMICs reported a mean estimate above 30%, and although most studies conducted in HICs were distributed in the lower end, 40% reported an annual expenditure of over 20% of the annual income. A study conducted in Canada among breast cancer patients estimated the lowest proportion of income spent as out-of-pocket costs at 2.3% [90]. At the other extreme, a study of pediatric cancer patients in India estimated that caregivers incurred considerable debt and spent over 175% of their annual income as medical and non-medical out-of-pocket costs [59]. However, this study had a small sample size and contributed relatively little to the estimated 42% weighted average income spent as out-of-pocket expenses in LMICs. Additionally, four studies defined explicit thresholds for catastrophic health expenditure [27,68,69,101]. They defined a threshold of annual income spent as out-of-pocket expenditures and estimated the proportion of patients exceeding it. In two studies, CHE was defined as 30% of the annual household income spent as non-reimbursed out-of-pocket costs in two studies conducted in different LMICs of South East Asia among an all cancer population [68,69]. This threshold was also defined at 40% in Haiti among breast cancer patients [101] and 10% in India among patients with pancreatic cancer [27]. The proportion of patients incurring CHE, however defined, ranged between 31% and 67%. 

Equity considerations and distributional effects were explicitly evaluated by a third of the included studies (*n* = 32). Three studies evaluated the out-of-pocket costs among different age groups; young adults and patients over 60 years of age faced comparatively higher out-of-pocket expenses [64,102,119]. On the other hand, two Australian studies and a study conducted in the U.S. estimated higher out-of-pocket costs among ethnic minorities and lower access to cancer care among indigenous populations [28,36,79]. Furthermore, four studies estimated additional out-of-pocket costs among patients living in rural and remote areas mostly due to increased expenses related to travel and transportation [44,51,65,109]. In settings with private insurance schemes, like in the U.S., patients with limited insurance packages paid higher deductibles and co-payments, especially for treatment and medications [27,41,48,60,73,97,98,104]. Finally, lower-income patients and households had a greater burden imposed by out-of-pocket expenses, as measured by the proportion of the household income spent in the form of out-of-pocket costs [31,34,47,68,69,74,86,87,90,92,108,116].

### Quality Assessment

**Figure 7:**
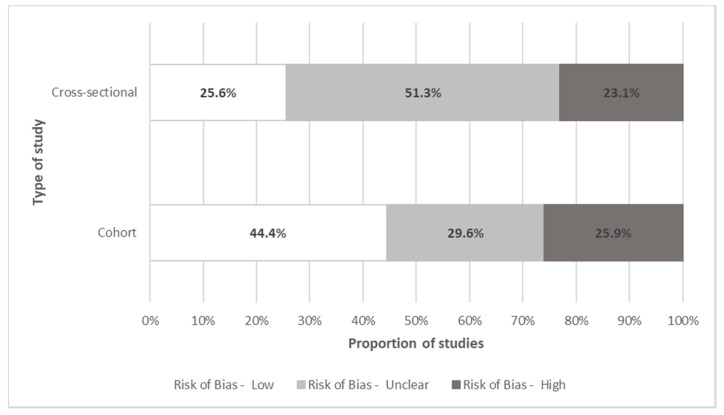
Quality assessment of individual studies. Note: This figure shows the proportion of studies with low, unclear or high risk of bias, as per the Ottawa-Newcastle Assessment Tool for cohort and cross-sectional studies. The dimensions evaluated for risk of bias were patient selection, comparability, and outcome assessment.

Risk of bias was assessed and summarized separately for cohort and cross-sectional studies (Figure 7). Forty-four percent of prospective cohort studies had a low risk of bias. Studies with unclear and high risk of bias mainly depended on self-reported out-of-pocket costs that patients recorded in their cost diaries but lacked verification (e.g., bills or receipts). Furthermore, cohort studies with unclear and high risk-of-bias usually failed to include a non-exposed cohort or failed to account for important confounders such as the type of insurance and income level across patients and households. On the other hand, 25% of cross-sectional studies had a low risk of bias. Most studies with unclear or high risk of bias failed to explicitly include a representative or random sample, or to account for important risk factors, effect modifiers or confounders.

## 4. Discussion

To the authors’ knowledge, this is the first systematic review to summarize and synthesize the existing literature on the out-of-pocket burden faced by patients diagnosed with cancer and their caregivers. This review found cancer patients pay substantial out-of-pocket costs per month, most of which is spent on cancer medications, followed by caregiver expenses, and transport and travel expenses. Expenditures were highest among pediatric patients and their caregivers. Furthermore, the out-of-pocket cost burden was comparatively higher in LMIC countries, and among underserved populations, such as ethnic minorities, populations living in rural and remote areas, and low-income patients and caregivers. This trend was seen across various studies conducted in different countries. An important finding was that patients incurred substantial out-of-pocket expenses (especially non-medical costs) in countries with systems that provide universal healthcare coverage, such as Canada, France, the UK, and Australia. 

The burden of paying out-of-pocket for medical care is a consequence of the varying degrees of comprehensiveness of public financing of cancer care in each setting. As an example, studies from countries that lack national insurance programs to cover essential medicines for the whole population (e.g., U.S.), usually reported high medication costs. This is further complicated by increasing costs of newer cancer-related medications that are usually covered by private insurance with considerable copayments [123]. However, although rising medication costs and their burden to the health care system remain an issue, this review focused primarily on costs incurred by patients/households. Patients also incurred substantial costs related to clinical consultations and in-hospital care (e.g., surgery) in HICs. In the U.S., these costs were likely an underestimate as the largest studies employed administrative datasets that included patients and caregivers with public and some private insurance. On the other hand, countries with universal healthcare coverage registered similar levels of expenditure for these categories, even though most of these procedures are considered medically necessary and are usually publicly funded. 

Paying out-of-pocket for essential cancer-related medicines and medical care results in high and potentially, catastrophic, levels of expenditure for cancer patients and their households. This can lead to cancer care becoming unaffordable in settings where there is sub-optimal health insurance coverage as patients and families are responsible for carrying a large portion of the cost burden of care. This poses a financial barrier to accessing cancer care that can impact on whether patients can adhere to their treatment plans. In other cases, patients opt for sub-standard care (e.g., cheaper and less effective IV therapies instead of expensive oral medications) due to the associated high deductibles and copayments [123]. Copayments have an impact on health service utilization rates as patients are often not well-positioned to distinguish between care that is necessary and care that might otherwise be defined as unnecessary. Reductions in unnecessary care are often overshadowed by reductions in overall health service use as well as changes in provider behaviour that are responsive to the patient-related reductions in utilization due to price; both of which can impact on health outcomes [124,125]. This review reinforces the importance of ensuring that essential cancer treatments are included in all healthcare benefit packages that are being developed to support achieving universal healthcare coverage, including in countries that are further along in the development and implementation of national health insurance programs.

This review also identified substantial expenditures for transport/travel (usually reported together in the studies) and caregiving, which are important for enabling access to and use of cancer treatment. However, support for these types of non-medical out-of-pocket costs tends to be inconsistent and varied [65,76,93,109]; as a result, we found non-medical costs were a key component of the overall out-of-pocket cost burden faced by patients across all studies in this review. Furthermore, non-medical costs may be under-reported, considering that most studies were conducted using employer-based administrative datasets that usually fail to capture this dimension. As such, health financing policies should be supplemented with a strengthening of social support programs to better recognize and address the significant burden associated with non-medical out-of-pocket costs. There may be opportunities to indirectly address the burden associated with some of the non-medical out-of-pocket costs as new models of community-based cancer care are developed and implemented. For example, the integration of virtual care and telemedicine into routine care could help ease the burden associated with travel and transport costs and potentially decrease some of the caregiver time and support required [126]. Similarly, interventions that integrate palliative and end-of-life care in the home [127] also have the potential to reduce caregiver and travel-related costs (e.g., lodging, food, fuel, etc.). In making future decisions about new models of cancer control, decision-makers should consider information on the full spectrum of costs and benefits associated with these programs, including their potential to mitigate the burden posed by out-of-pocket costs.

The economic burden associated with cancer due to out-of-pocket spending has been more recently described as financial toxicity because of the impact that it has on the economic circumstances of households [128]. Previous systematic reviews found that financial toxicity was common among cancer survivors, partly due to the high out-of-pocket costs associated with their cancer care. However, these studies highlighted a lack of information regarding at-risk populations and intervention targets that would allow developing interventions capable of mitigating financial toxicity among cancer patients and their caregivers [129,130]. As such, this review confirms some populations are consistently more at risk of facing financial toxicity associated with cancer. Pediatric patients and their caregivers experienced considerably higher out-of-pocket costs mainly due to relatively longer and more resource-intensive treatment and costly survivorship care [131]. In particular, LMICs in general, and lower-income households (in both LMICs and HICs) were more heavily burdened and experienced financial toxicity more frequently. For example, low-income households with pediatric cancer patients in India paid more than twice their monthly earnings to cover the associated out-of-pocket expenses, thus incurring considerable debt [59]. This trend was also observed among patients who were unemployed and those who lacked or did not have private health insurance [27,41,48,60,73,97,98,104]. Some ethnic minorities and Indigenous communities, who often reside in rural and remote areas, experienced higher levels of out-of-pocket costs in Australia—other communities reported no costs due to a reduced, and almost non-existent access to health care [28,36]. These risk factors are not independent; most vulnerable populations often face multiple barriers to healthcare and an increasingly larger out-of-pocket burden. These are pressure points that healthcare and social care systems should seek to address to minimize the burden for patients and their caregivers, in particular those sub-groups who are most at risk of falling through the cracks [132]. 

This review makes an important contribution to the literature by estimating the magnitude and distribution of non-reimbursed costs that specific cancer populations face in different contexts; nonetheless, there are a few limitations. Our literature search focused on studies retrieved from only four databases; nonetheless, and based on prior reviews, these are the most relevant databases given the topic [129,133]. We reported and extracted an average cost across all included studies; however, the cancer populations examined and cost definitions were heterogeneous. Furthermore, not enough information was available to pool costs across countries and cancer types, or to estimate total out-of-pocket costs per treatment. Therefore, an overall estimate might not appropriately describe the distribution of out-of-pocket costs in all settings. Consequently, we estimated an average cost across spending categories, cancer sites, and different countries, to better understand how these costs were distributed among different populations and country income levels. Although a distinction between costs borne by cancer patients and their caregivers was of interest, it was not possible to explore this due to lack of individual category estimates. However, the ‘caregiver cost’ category provided an estimate of how much was spent on supportive care, daycare for pediatric patients, and other formal and informal care provided by caregivers. Furthermore, this review likely provides an underestimate of the out-of-pocket cost burden for cancer patients and their caregivers; many studies focused on single cost categories (e.g., medications), instead of evaluating multiple types of non-reimbursable expenditures. The review was also limited by the lack of evidence from LMICs. Most studies did not include enough information to allow estimating a weighted average. Additionally, presenting country-specific out-of-pocket costs stratified by cancer site and expenditure categories would have allowed for a direct comparison of more heterogeneous populations. However, in most cases, the sample size was only large enough to do so for the U.S. Finally, only one-third of the studies provided enough information to estimate the out-of-pocket cost burden. Absolute measures (i.e., total out-of-pocket cost) provide information regarding how much patients and caregivers are spending on cancer care but fail to account for the burden of this expenditure on the household’s resources. On the other hand, a relative measure such as the proportion of income spent on out-of-pocket medical costs allows an understanding of how a household might be burdened by these expenses;, e.g., a higher proportion is usually associated with financial debt and a reduction of spending on food, rent, clothes, and education [68,69,98]. Studies should seek to employ a consistent approach to measure the out-of-pocket burden as an absolute and a relative measure to allow for comparisons across heterogeneous jurisdictions and populations. Moreover, although the existing literature mostly focuses on the indirect and out-of-pocket burden of cancer, further studies should evaluate the relationships between out-of-pocket costs and the psychosocial burden of cancer. Studies have found that patients and caregivers who incur catastrophic health expenditures can experience financial strain and distress, which can contribute to the psychosocial burden [134,135]. Consequently, out-of-pocket costs not only pose a burden in terms of costs and potential nonadherence to treatment but might also affect patients’ quality of life. To fully understand this inter-relationship and the extent to which out-of-pocket costs contribute to the overall burden of cancer, the relationship between its components must be described. 

This review supplements the growing body of literature on the economic burden of cancer for patients and their caregivers. It builds on this work by providing estimates of the out-of-pocket costs associated with cancer care and explores whether there is consistency in this burden across cancer populations and settings. The results of this study are an important input for advancing the agenda of addressing financial toxicity [128] as it provides estimates of how much patients pay for their cancer care while highlighting pressure points in the overall financing of cancer treatment across settings. Furthermore, this review confirms that patients are still key funders of cancer treatment in many countries, including in systems with universal healthcare coverage, despite varying abilities to afford these costs. The results also suggest the need for comprehensive out-of-pocket costing data for different cancer sites, and patient and caregiver populations across the cancer care continuum to inform planning and decision making. This review will help support planning and decision-making discussions, which should ensure that the economic burden on patients and families is accounted for when setting priorities to sustain the cancer care system.

## Figures and Tables

**Table 1:** Study characteristics.

First Author	Year	Country	Cancer Care Continuum	Sample Size	Mean Age (SD)	% over 60 Years	% Female	% Insured	Type of Insurance	Mean	Study Design	Study Population
										Income		
**All cancer (adults)**												
Bates	2018	Australia	Post diagnosis	25,530	NR	57.20%	44	NR	NR	NR	Retrospective observational study	All cancer patients in Queensland
Callander	2019	Australia	Post diagnosis	429	57.4 (15.4)	NR	49	100%	Public	NR	Retrospective observational study	All cancer patients in Queensland
Gordon	2009	Australia	Diagnosis onwards	439	57 (12)	NR	61	47%	Private	55% of households earned < AUD 40,000 per year	Cross-sectional	Adults diagnosed or treated for cancer at the Townsville Hospital Cancer Centre
Newton	2018	Australia	Diagnosis	400	64 (11)	53% over 65	49	100%	Any	AUD 919 weekly per household	Cross-sectional	Cancer patients who resided in rural regions of Western Australia
Action Study Group	2015	Cambodia, Indonesia, Laos, Malaysia, Myanmar, Philippines, Thailand, Vietnam	Treatment (post-surgery)	4584	51	13% over 65	72	44%	Any	NR	Prospective cohort study	All cancer patients with planned surgery
Yu	2015	Canada	Palliative care	186	NR	61% over 70	54.84	100%	Public	NR	Cohort	End of life
Dumont	2015	Canada	Palliative care	252	58	NR	73	100%	Public	NR	Longitudinal, prospective design with repeated measures	Patients enrolled in a regional palliative care program and their main informal caregivers
Longo	2011	Canada	Treatment onwards	282	61.6	NR	47	100%	Public	11% of households earned < USD 20,000 CAD per year	Cross-sectional design	Urban and rural patients in 5 of the 8 cancer clinics in the province of Ontario
Wenhui	2017	China	Treatment	2091	63	NR	NR	100%	Any	NR	Cohort	NR
Koskinen	2019	Finland	Diagnosis onwards	1978	66 (26–96 range)	NR	45	100%	Public	NR	Cross-sectional registry and survey study	Patients having either prostate, breast or colorectal cancer
Buttner	2018	Germany	Treatment onwards	502	46 (8)	0%	46.6	100%	Any	33.3% households earned <USD 1000 Euros per year	Prospective cohort study	Working age cancer patients
Mahal	2013	India	Diagnosis onwards	821	NR	NR	NR	3.41%	Private	NR	Cross-sectional survey	Household with at least one person living with cancer, or hospitalized due to cancer
Collins	2017	Ireland	Treatment onwards	151	Median 58 (range 20–79)	NR	60	NR	NR	NR	Retrospective cohort study	Cancer patients, 18 years or older
Baili	2015	Italy	Survivorship	296	NR	17% over 80	59	NR	NR	NR	Retrospective cohort study	Patients diagnosed between 2003 and 2007
Isshiki	2014	Japan	Treatment	521	63	NR	NR	NR	NR	NR	Observational descriptive study	Cancer patients receiving anti-cancer treatment
Action Study Group	2016	Singapore, Brunei, Malaysia, Thailand, Indonesia, Philippines, Vietnam, Laos, Cambodia, Myanmar	Diagnosis onwards	9513	Median 52 (IQR = 26)	NR	37	43	Any	41% of households earn 0–75% of mean national income	Prospective cohort	Newly diagnosed adult cancer patients recruited from 47 public and private hospitals
Marti	2015	UK	Survivorship	298	NR	56%	55	100%	Public	NR	Prospective cohort study	Patients diagnosed with potentially curable breast, colorectal or prostate cancer
Bernard	2011	USA	Treatment onwards	4110	NR	43% over 55	62	94%	Any	USD 62,026 year in 2004 per household	Case–control	Persons 18 to 64 years of age who received treatment for cancer
Chino	2018	USA	Treatment	245	Median 60—Range 27–91	NR	55	100%	Any	NR	Retrospective cohort study	Patients with solid tumour cancers receiving chemotherapy or hormonal therapy
Colby	2011	USA	Treatment	329	NR	NR	94.9	100%	Public	NR	Retrospective cohort study	Cancer patients who discontinued medication
Davidoff	2012	USA	Diagnosis onwards	1868	NR	94% over 65	49	100%	Public	USD 35,356 per year per patient	Retrospective, observational study	Medicare beneficiaries with newly diagnosed cancer
Dusetzina	2017	USA	Treatment	63,780	NR	NR	57.2	100%	Any	NR	Retrospective, observational study	Patients aged 18 through 64 years who had prescription drug coverage
Dusetzina	2016	USA	Treatment	3344	NR	NR	NR	100%	Any	NR	Retrospective, observational study	Orally administered anticancer medications
Finkelstein	2009	USA	Treatment onwards	679	50 (10.1)	NR	69	79.80%	Any	USD 49,240 per year per household	Retrospective, observational study	Working age cancer patients (age 25–64)
Guy	2018	USA	Diagnosis onwards	4271	NR	65% over 50	65	89.30%	Any	NR	Retrospective observational study	Adults with a cancer diagnosis
Houts	1984	USA	Treatment	139	57	NR	66	NR	NR	NR	Prospective observational study	Patients receiving outpatient chemotherapy treatments in seven oncology practices
John	2016	USA	Survivorship	2977	61.9 (0.8)	44.7% over 65	48	94%	Any	NR	Cross-sectional	Adults who self-reported ever having received any cancer diagnosis
Kircher	2014	USA	Treatment	6607	70.1 (0.2)	NR	48	100%	Any	NR	Case–control	Individuals aged over 55 years with cancer coded in the condition file in MEPS
Langa	2004	USA	Treatment onwards	988	80 (0.3)	100%	54	100%	Public	NR	Observational descriptive study	Cancer patients over 70 years old
Narang	2016	USA	Diagnosis onwards	1409	Median 73 (IQR 69–79)	NR	46.4	100%	Any	25% of households earned < USD 22,380 per year	Prospective cohort study	US residents older than 50 years
Raborn	2012	USA	Treatment	6094	53 (13)	NR	54.4	100%	Any	NR	A retrospective claims-based analysis	Patients over 18 years with at least one claim of an oral oncolytic therapy
Shih	2015	USA	Treatment	200,168	52	NR	NR	100%	Private	NR	Cohort	Patients undergoing chemotherapy, in Lifelink Health Plan Claims Database
Shih	2017	USA	Treatment	42,111	72.17 (9.93)	100% over 65	50.9	100%	Private	NR	Cohort	Medicare beneficiaries, insured
Stommel	1992	USA	Treatment	192	58.7 (12.2)	NR	49.5	NR	NR	USD 34,473 per household per year	Cross-sectional	Study sample had at least one dependent in an activity of daily living and caregiver
Tangka	2010	USA	NR	24,654	NR	NR	NR	100%	Any	NR	Cohort	Panel survey population
Tomic	2013	USA	Treatment	28,979	59 (12)	29% over 65	71	100%	Any	NR	Cohort	Adult patients who received chemotherapy and granulocyte colony-stimulating factors in the outpatient setting in the United States
Kaisaeng	2014	USA	Treatment	3781	75 (7)	NR	97	100%	Public	NR	Cross-sectional	Medicare beneficiaries who filled a prescription for imatinib, erlotinib, anastrozole, letrozole, or thalidomide during 2008.
Markman	2010	USA	NR	1767	NR	42%	58	NR	NR	7% of households earned < USD 20,000 per year	Observational descriptive study	Breast, colon, lung, and prostate cancer who joined the NexCura program
Jung	2018	USA	Treatment	148,265	76 (7.3)	NR	51	100%	Public	USD 61,317 per year	Natural experimental design	Elderly Medicare beneficiaries with cancer
Chang	2004	USA	Diagnosis onwards	58	67 (12)	NR	30	100%	Any	NR	Retrospective matched-cohort control	Individuals insured by private or Medicare supplemental health plans
**All cancer (pediatric)**												
Cohn	2003	Australia	Diagnosis onwards	100	8.9 (range 0.8–18)	0%	50	100%	Any	NR	Cross-sectional	Children with cancer and their families
Tsimicalis	2013	Canada	Treatment	78	37.38 (parents)	NR	NR	100%	Public	Assumed: USD 73,500 per household per year	Cohort, cost of illness	Convenience sample
Tsimicalis	2012	Canada	Treatment	99	7.85 (5.28)	NR	NR	100%	Public	Assumed: USD 73,500 per household per year	Cohort, cost of illness	Convenience sample
Ahuja	2019	India	Diagnosis onwards	11	NR	NR	NR	NR	NR	NR	Prospective cohort	Children with cancer and their families
Sneha	2017	India	Treatment	70	7.8 (2.2)	0%	31	0%	Private	15% of households earned< 60,000 Rs. per year	Cross-sectional	Clinical setting
Ghatak	2016	India	Treatment onwards	50	5.6 (2.9)	0	24	NR	NR	239 USD per month per household	Prospective observational study	Families with children with acute lymphoblastic leukemia
Bloom	1985	USA	Diagnosis onwards	569	NR	NR	NR	NR	NR	USD 25,790 annual family income	Retrospective observational study	Children with malignant neoplasms
Lansky	1979	USA	Treatment	70	7 (4.5)	0	34	NR	NR	USD 13,500	Prospective cohort study	Parents of children in treatment for cancer by the pediatric hematology department
**Breast**												
Boyages	2016	Australia	Treatment	361	NR	56% over 55	100	NR	NR	20.6% households earned < USD 45,000 (AUD 2016) per year	Cross-sectional	Females with primary stage I, II, or III breast cancer; had completed treatment at least 1 year prior to recruitment; and fluent in English
Gordon	2007	Australia	Diagnosis onwards	287	57 (9.6)	62% over 50	100	70%	Private	29% of patients earned < USD 26,000 AUD per year	Longitudinal, population-based study	Women with breast cancer 0–18 months post-diagnosis
Housser	2013	Canada	Treatment onwards	301	NR	47% over 65	43	64.60%	Private	14.1% of patients earned less than CAD 20,000 per year	Observational descriptive study	19 years of age or older, residents of Newfoundland, and diagnosed with breast or prostate cancer
Lauzier	2012	Canada	Diagnosis-treatment	1191	NR	31.60%	67	100%	Public	58% of households earned < USD 50,000 per year	Prospective cohort study	Women with breast cancer and their spouses
Liao	2017	China	Diagnosis-treatment	2746	49.6	7% over 65	100	100%	Any	USD 8722	Multicentre cross-sectional study	Patients with breast cancer diagnosis at a hospital affiliated with the CanSPUC project
O’Neill	2015	Haiti	Diagnosis onwards	61	49 (9.8)	NR	98	NR	NR	USD 1333 per year per patient	Cross-sectional	Patients from Hopital Universitaire de Mirebalais
Bargallo-Rocha	2017	Mexico	Treatment	69	Median 56 (IQR 11.5)	NR	100	NR	NR	USD 548 in Mexico/ month	Cross-sectional	Female patients who underwent breast cancer surgery
Bekelman	2014	USA	Treatment	15,643	NR	34.2	100	100%	Private	13.5% households earned <USD 40,000/year	Retrospective observational study	Women with breast cancer with breast conserving surgery
Chin	2018	USA	Treatment	6900	NR	21%	100	100%	Public	NR	Retrospective cohort study	Female patients aged 18 to 64 years
Dean	2018	USA	Survivorship	129	65 (8)	NR	100	98%	Any	13% patients earned <USD 30,000 per year	Prospective, longitudinal study	Women with stages I–III invasive breast cancer, completion of active breast cancer treatment, > 1 lymph node removed
Farias	2018	USA	Treatment	6863	NR	17.30%	100	100%	Private	NR	Retrospective, observational study	Women under the age of 64 with at least 1 prescription claim
Giordano	2016	USA	Diagnosis onwards	14,643	Median 54	12.2% over 65	100	100%	Any	NR	Observational Cohort Study	Women aged over 18 years with breast cancer diagnosed between 2008 and 2012
Jagsi	2014	USA	Diagnosis onwards	1502	NR	28% over 65	100	NR	NR	USD 50,000 per year	Longitudinal cohort study	Patients age 20 to 79 years diagnosed with stage 0 to III breast cancer
Jagsi	2018	USA	Diagnosis onwards	2502	NR	57%	100	95%	Any	37% of households earned < USD 40,000 per year	Cross-sectional survey	Patients with early stage breast cancer
Leopold	2018	USA	Treatment—end of life	5364	NR	58% over 50	100	100%	Any	USD 50,054	Longitudinal time series	Insured women with metastatic breast cancer
Pisu	2016	USA	Survivorship	432	NR	47.7% over 65	100	94%	Public	19.3% lowest income (<20,000 per year)	Prospective cohort study	Stage 0–III breast cancer, within the first three years after completing primary cancer treatment
Pisu	2011	USA	Survivorship	261	NR	16% over 65	100	NR	NR	11.5% lowest income (<20,000 per year)	Cross-sectional	Patients diagnosed with stage I–II breast cancer, with a minimum of 1 month after treatment completion
Roberts	2015	USA	Treatment	18,575	53.6 (7.5)	NR	100	100%	Private	NR	A retrospective claims-based analysis	Women (ages 18–64) with at least two health encounter claims for breast cancer
**Leukemia**												
Kodama	2012	Japan	Treatment	577	Median 61 (15–94 range)	NR	35	100%	Public	USD 36,731 USD per year	Observational descriptive study	Patients with CML who were prescribed imatinib
Wang	2014	Singapore	Treatment	367	NR	8% over 61	62.1	NR	NR	NR	Cohort	Secondary analysis of a prospective study
Darkow	2012	USA	Treatment	995	62	NR	47	100%	Any	NR	Retrospective cohort study	Adult patients (aged >18 years) with an initial diagnosis of CML during 1997 to 2009
Doshi	2016	USA	Diagnosis onwards	1053	73 (8)	96% over 65	47	100%	Public	NR	A retrospective claims-based analysis	Medicare patients with newly diagnosed CML
Dusetzina	2014	USA	Treatment	1541	48(11)	NR	45	100%	Any	NR	Retrospective, observational study	Adults (age 18 to 64 years) with CML who initiated imatinib therapy
Goodwin	2013	USA	Treatment onwards	1015	61 (9.2)	NR	39	97%	Any	NR	Observational descriptive study	Patients who had received intensive treatment for MM at the study site
Gupta	2018	USA	Treatment	162	56 (13)	NR	49	97%	Any	42% patients earned less than USD 50,000 USD per year	Cross-sectional	Adult patients with MM taking medication
Shen	2017	USA	Treatment	898	70 (12)	NR	47	38%	Public	NR	Retrospective cohort study	Patients with Chronic Myeloid Leukemia Taking Targeted Oral Anticancer Medications
Olszewski	2017	USA	Treatment	3038	Median76 (IQR 71–82)	NR	50	100%	Public	USD 29,700 per year	Observational descriptive study	Patients with Part D coverage at diagnosis
**Colorectal**												
Huang	2017	China	Diagnosis onwards	2356	57.4	28.3% over 65	43	100%	Any	CNY 54,525 per patient per year	Cross-sectional survey	Primary prevalent CRC patients undergoing treatment in hospitals
Hanly	2013	Ireland	Diagnosis and treatment	154	NR	60% over 55	82	NR	NR	NR	Retrospective observational study	Carers of colorectal cancer patients
O Ceilleachair	2017	Ireland	Survivorship	497	67	46% over 70	38	52%	Private	NR	Case report	All cases of primary, invasive colorectal cancer in Ireland diagnosed October 2007–September 2009
Shiroiwa	2010	Japan	Treatment	1319	NR	NR	NR	100%	Public	NR	RCT, EE	Trial population, XELOX or XELOX plus bevacizumab and second-line therapy with XELOX
Azzani	2016	Malaysia	Diagnosis onwards	138	Median 63 (IQR = 19)	35.5% over 70	49	9%	Private	2000 RM/month	Prospective, longitudinal study	CRC patients seeking treatment at the University of Malaya Medical Centre (UMMC) in the first year following diagnosis
Sculpher	2000	UK	Treatment	495	61	NR	36	NR	NR	NR	Randomized-controlled trial	Colorectal cancer patients treated with Raltitrexed or Fluorouracil
**Lung**												
Ezeife	2018	Canada	Treatment—Palliative care	200	NR	50% over 65	56	45.10%	Private	USD 41,000-USD 80,000 CAD	Cross-sectional	Patients with advanced lung cancer (stage IIIB/IV)
Andreas	2018	France, Germany and the United Kingdom	Treatment onwards	831	NR	67%	38	100%	Public	NR	Retrospective observational study	Patients ≥18 years of age that had undergone complete resection of stage IB-IIIA NSCLC
Wood	2019	France, Germany, Italy	Treatment	1457	64.5 (10.1)	NR	34.1	NR	NR	NR	Cross-sectional	NR
Van Houtven	2010	USA	Initial treatment, Continuing, Terminal, overall	1629	NR	42.1% over 65	75.8	100%	Any	USD 39,554 per year per household	Cross-sectional	Informal caregivers—Patients participating in the Share Thoughts on Care survey
Hess	2017	USA	Diagnosis onwards	47,207	65 (10.4)	NR	45	100%	Any	NR	Retrospective observational study	18 years of age or older at the time of initial diagnosis of lung cancer
**Head and neck**												
Burns	2017	Australia	Survivorship-integrated care	82	65 (7.4)	NR	26	NR	NR	NR	Randomized-controlled trial	Patients with head and neck cancer enrolled in speech pathology programs
Chauhan	2018	India	Treatment	410	NR	NR	NR	NR	NR	NR	Retrospective observational study	Head and cancer patients undergoing radiotherapy
de Souza	2017	USA	Treatment onwards	73	60 (26–79)	NR	21.9	100%	Any	USD 81,597 per year per household	Prospective observational study	Head and neck cancer patients with locally advanced stage
Massa	2019	USA	Diagnosis onwards	16,771	65 (95CI 63.1–66.8)	NR	35.5	97.40%	Any	USD 24,056	Case control	Adult patients with cancer
**Prostate**												
Gordon	2015	Australia	Diagnosis onwards	289	65 (8.4)	78%	0	71%	Private	38% households had incomes between USD 37,000 and AUD 80,000 per year	Cross-sectional	Men who self-reported they had previously been diagnosed with prostate cancer
de Oliveira	2013	Canada	Survivorship	585	73	92.50%	0	100%	Public	40% earned <USD 40,000 per year	Retrospective, observational study	All patients initially diagnosed with PC in 1993–1994, 1997–1998, and 2001–2002
Geynisman	2018	USA	Treatment	116	65 (range 27–88)	NR	15	98%	Any	NR	Retrospective, observational study	Advanced renal and prostate cancer patients
Jayadevappa	2010	USA	Diagnosis—Treatment	512	59 (6.3)	NR	0	NR	NR	19% of patients earned < USD 40,000 per year	Prospective cohort study	45 years of age, newly diagnosed with PCa within the prior 4 months and yet to initiate\ treatment
**Skin**												
Gordon	2018	Australia	Treatment onwards	419	55	NR	54	74%	Private	NR	Retrospective, observational study	Consenting Qskin study participants
Gordon	2018	Australia	Treatment onwards	539	56	NR	64	NR	NR	NR	Retrospective, observational study	Consenting Qskin study participants
Thompson	2019	Australia	Treatment	8613	NR	NR	NR	100%	Public	NR	Cohort	Admin data linked to study population
Grange	2017	France, Germany and the United Kingdom	Survivorship and Palliative care	558	NR	54.50%	44	100%	Public	NR	Retrospective observational study	Patients with advanced melanoma
**Ovarian**												
Bercow	2018	USA	Diagnosis onwards	5031	NR	41.40%	100	100%	Private	NR	Retrospective cohort study	Ovarian cancer patients enrolled in commercial insurance sponsored by over 100 employers in the United States
Calhoun	2001	USA	Treatment	83	NR	NR	100	NR	NR	NR	Prospective cohort study	Ovarian cancer patients who experienced chemotherapy-associated hematologic or neurologic toxicities
Suidan	2019	USA	Treatment	12,761	NR	44%	100	100%	Private	NR	Cohort	All ovarian cancer patients in MartketScan database undergoing first line treatment
**Pancreatic**												
Basavaiah	2018	India	Treatment	98	54.5 (10–87 range)	41.8% over 60	33	29.60%	Any	NR	Prospective cohort	Patients undergoing pancreatic-duodenectomy
Bao	2018	USA	End-of-life	3825	NR	100%	55	100%	Public	NR	Retrospective cohort study	Patients 66 years or older when diagnosed with Stage IV pancreatic cancer in 2006–2011
**Anal**												
Chin	2017	USA	Treatment	1025	NR	NR	65	100%	Public	NR	Retrospective cohort study	Patients with anal cancer treated with Intensity-modulated radiotherapy
**Brain**												
Kumthekar	2014	USA	Treatment	43	Median 57 (range 24–73)	NR	42	95%	Any	USD 75,000 per year	Prospective observational study	Patients within 6 months of diagnosis or tumor recurrence

AUD = Australian dollar; CML = chronic myeloid leukemia; CNY = Chinese Yuan; NSCLC = Non-small-cell lung carcinoma; IQR = interquartile range; MM = multiple myeloma; NR = not reported; RM = Renminbi; Rs. Rupees; SD = standard deviation; USD = United States Dollar.

**Table 2:** Out-of-pocket estimates.

First Author	Year	Definition of out-of-Pocket Cost	Total out-of-Pocket Cost Estimate (Mean—SD)	Time Frame of out-of-Pocket Estimate	Out-of-Pocket as % of Income	Currency	Currency Year
The Action Study Group	2016	Financial catastrophe was defined as OOP costs at 12 months exceeding 30% of annual household income	NR	3- and 12-month follow-ups	48% of cancer patients reported Financial catastrophe at 12 months	NR	NR
The Action Study Group	2015	Financial catastrophe (out-of-pocket costs of >30% of annual household income)	NR	3 months	31% of participants incurred Financial catastrophe	NR	2015
Ahuja	2019	Direct costs incurred by families of children being treated for cancer	651 (356)	14 weeks	NR	USD	2013
Andreas	2018	Cost of childcare, and non-reimbursed transportation costs incurred by the patient or their family/friends.	UK = 7	1 month	NR	Euro	2013
			Germany = 6				
			France = 0				
Azzani	2016	Payments for expenses such as hospital stays, tests, treatment, travel and food.	8306	1 year	42% of the median annual income in Malaysia.	RM	2013
Baili	2015	Direct expenses which were not entirely covered or only partially covered by the NHS	160 (372)	1 month	NR	Euro	2015
Bao	2018	Costs incurred by patients 30 days before death	1930 (with chemotherapy)	1 month	NR	USD	2011
Bargallo-Rocha	2017	Patient borne costs on transportation, housing, and salary due to breast cancer	535	1 month	NR	USD	2017
Basavaiah	2018	Catastrophic expenditure was defined as the percentage of households in which OOP health payments exceeded 10% of the total household income	NR	From the first hospital visit to postoperative recovery	A total of 76.5% of the sample incurred catastrophic expenditure	USD	2015
Bates	2018	Patient co-payments for primary healthcare and prescription pharmaceuticals	1000 (2000)	1 year	NR	AUD	2017
Bekelman	2014	Summing deductible, co-payment, and coinsurance amounts.	3421 (95% CI 3158–3706)	1 year	NR	USD	2013
Bercow	2018	Out-of-pocket (OOP) payment was calculated as the sum of deductibles, copayments, and coinsurance	Median 2988 (IQR 1649–5088)	1 year	NR	USD	2013
BerNRrd	2011	OOP expenditures on health insurance premiums in addition to OOP expenditures on healthcare services	4772	NR	6%	USD	2008
Bloom	1985	Direct medical and nonmedical expenses borne by the family	9787	1 year	37.7% of family income	USD	1981
Boyages	2016	The financial cost of lymphedema care borne by women	977	1 year	NR	AUD	2014
Burns	2017	Costs associated with return travel to the regional speech pathology service	256	NR	NR	AUD	2015
Buttner	2018	Direct payments for health services or treatments which are not covered by health insurance and need to be paid by the patients themselves	205 (346)	3 months	NR	Euro	2018
Calhoun	2001	Direct medical costs borne by patients	3302	3 months	NR	USD	2001
Callander	2019	Patient co-payments for primary healthcare and prescription pharmaceuticals	1191 (3099)	1 year	NR	AUD	2017
Chang	2004	Copays and deductibles to caregivers	302 (634)	1 month	NR	USD	2004
Chauhan	2018	Only the direct OOP expenditure was assessed	849	NR	NR	USD	2015
Chin	2018	Copayments for oral anticancer medication	19	1 month	NR	USD	2014
Chin	2017	The sum of Medicare Part A and Part B reimbursements, third-party payer reimbursements, and patient liability amounts	Median 6967 (5226–9076)	1 year	NR	USD	2011
Chino	2018	Insurance premiums; medication copays; physician office charges; copays for procedures, tests, and studies; and costs related to travel for treatment	Median 393 (Range—0–26,586)	1 month	7.80%	USD	2018
Cohn	2003	Travel, accommodation, and communication costs	19,604 (32,976)	40 months	NR	AUD	2003
Colby	2011	Patient spending on ani cancer drugs	645	3 months	NR	USD	2011
Collins	2017	Personal expenditure on regular and non-regular indirect costs during treatment.	1138 (range 21–7089)	1 month	NR	EUR	2017
Darkow	2012	Copayment for anti cancer medication	124	1 month	NR	USD	2012
Davidoff	2012	Costs incurred by patients	4727 (202)	2 years	23.90%	USD	2007
de Oliveira	2013	Medical costs associated with health Professional visits, and nonmedical costs such as travel, parking, food, and accommodation	200 (95% CI USD 109–290)	1 year	10%	CAD	2006
de Souza	2017	Insurance premiums; deductibles; direct medical costs	805 (range 6–10,156)	1 month	15.10%	USD	2017
Dean	2018	Co-payments for outpatient physician visits, physical and occupational therapy visits, complementary and integrative therapy visits	2306	1 year	NR	USD	2015
Doshi	2016	Direct medical costs borne by patients	2600	1 month	NR	NR	2016
Dumont	2015	NR	576 (46)	6 months	NR	CAD	2015
Dusetzina	2017	Copayment, coinsurance, and deductibles, adjusting to reflect spending on a median monthly dosage	143	1 month	NR	USD	2012
Dusetzina	2016	Copayments for orally administered anticancer medications	310	1 month	NR	USD	2014
Dusetzina	2014	Monthly copayments for imatinib	108 (301)	1 month	NR	USD	2011
Ezeife	2018	Expenses for prescription drugs, travel, childcare/babysitting, copayments, and deductibles	Median 1000–5000	1 year	From 2–12% (median)	CAD	2018
Farias	2018	Sum of the copayments, deductibles, and coinsurance paid for AET medication	193 (97)	1 month	NR	USD	2018
Finkelstein	2009	Copayments, deductibles, and payments for noncovered services	1730 (2710)	1 year	NR	USD	2005
Geynisman	2018	Co-pays for oral anti cancer medications	81.26	1 month	NR	USD	2018
Ghatak	2016	Direct medical, living (rent, food, clothes), and transport costs	Median 524 (395–777 IQR)	1 month	3.5 times–7 times the monthly income	USD	2013
Giordano	2016	Drug and insurance-related costs borne by patients	3226	18 months	NR	USD	2013
Goodwin	2013	Direct and indirect patient expenditure	NR	1 year	38% and 31% annually for patients receiving/not receiving chemotherapy, respectively.	NR	2013
Gordon	2007	Direct costs (garments and aids), health services (e.g., co-payments, pharmaceuticals) and paid home services	1937 (3210)	18 months	NR	USD	2005
Gordon	2018	Melanoma treatment costs borne by patients	625 (575)	3 years	NR	AUD	2016
Gordon	2018	Medical expenses for Medicare services borne by patients	3514 (4325)	2 years	NR	AUD	2016
Gordon	2009	Medical and non-medical costs borne by patients	4826 (5852)	16 months	NR	AUD	2008
Gordon	2015	Medical and non-medical costs borne by patients	9205 (14,567)	16 months	NR	AUD	2012
Grange	2017	Childcare and non-reimbursed transportation costs	France = 0	1 month	NR	EUR	2013
			Germany = 332 (95% CI 271–401)				
			UK = 533 (477–594)				
Gupta	2018	Costs for doctor visits, prescriptions, over-the-counter medications, transportation	709 (1307)	3 months	NR	USD	2018
Guy	2018	Expenditures toward any healthcare service, such as coinsurance, copayments, and deductibles	2171 (95% CI 1970–2373)	1 year	4.3% had OOP > 20% of household income	USD	2012
Hanly	2013	Parking, meals and accommodation, domestic-related caring activities	79.2 (151)	1 week	NR	EUR	2008
Hess	2017	Copayments, deductibles and patient borne costs	315 (95% CI 106–523)	1 month	NR	USD	2014
Housser	2013	Costs not covered by insurance or assistance programs	Prostate: 910 (1025) Breast: 864 (1220)	3 months	17% had OOPC >7.5% of income (16% prostate, 19% breast)	CAD	2008
Houts	1984	Nonmedical expenses borne by patients	Median 21 (0–204 range)	1 week	28% of respondents were spending over 25% of their weekly incomes	USD	1984
Huang	2017	Overall medical and non-medical expenditure	32,649	1 year	59.9% of their previous-year household income	CNY	2014
Isshiki	2014	Travel/transport costs per outpatient treatment	79	1 month	NR	USD	2014
Jagsi	2014	Medical expenses related to breast cancer, including copayments, hospital bills, and medication costs	Median <2000	4 years	NR	USD	2014
Jagsi	2018	Medical and non-medical expenses related to breast cancer (including copayments, hospital bills, and medication costs)	Median <2000	NR	17% of patients reported spending ≥10% of household income on out-of-pocket medical expenses	USD	2018
Jayadevappa	2010	Medication and non-medical costs paid by patients	703 (2500)	3 months	NR	USD	2010
Jung	2018	Costs of specialty cancer drugs paid by patients	3860 (1699)	1 year	NR	USD	2013
John	2016	Alternative medicine costs borne by patients and not covered by insurance	445	1 year	NR	USD	2012
Kaisaeng	2014	Copayments for oral anti cancer drugs	154 (407)	1 month	NR	USD	2008
Kircher	2014	Direct payment for all prescription drugs	724 (42)	NR	NR	USD	2010
Kodama	2012	Copayment for medical expenses	Median 11,548	1 year	NR	USD	2008
Koskinen	2019	Out of pocket fees for outpatient visits, inpatient care, home care, and surgical procedures	280 (603 for palliative care—383 metastatic disease—224 remission—264 rehabilitation—263 treatment)	6 months	NR	Euro	2010
Kumthekar	2014	Medical and nonmedical expenses that were not reimbursed by insurance	2451 (2521)	1 month	NR	USD	2014
Langa	2004	Cost paid by patients on hospital services, outpatient care, home care, and medication	4656 (3890)	1 year	NR	USD	1995
Lansky	1979	Non-medical costs paid by the patient’s family	56 (54)	1 week	26% of weekly income	USD	1979
Lauzier	2012	Costs for treatments and follow-up, consultations with other practitioners, home help, clothing, and natural health products	1365 (1238)	1 year	Out-of-pocket costs represented an average of 2.3% of annual family income	CAD	2003
Leopold	2018	Patient expenditures including coinsurance, copayment, and deductible amounts	4247—95% CI (3956–4538) among low deductible health plan	1 year	13% of the 2011 real median income household	USD	2012
			6642—95% CI (6268–7016) among High deductible health plan				
Liao	2017	Medical expenditure (self-pay and healthcare costs), non-medical expenditure (i.e., transportation, accommodation)	8449	Since diagnosis to treatment	49% (overall OOP expenditure/annual income)	USD	2014
Longo	2011	Patient borne costs	Breast 392 (830)	1 month	NR	CAD	2001
			Other 149 (265)				
Mahal	2013	Patient medical and non-medical spending	5311 (4514–6108 95CI)	1 year	NR	INR	2004
Markman	2010	Cancer related costs paid by patients	12% spent between USD 10,000–25,000	Since diagnosis	NR	USD	2010
			4% spent between USD 25,000–50,000				
			2% spent between USD 50,000–100,000				
Marti	2015	Medical and non-medical costs borne by patients, such as medications, travel, and childcare	Full Sample 39.8 (95% CI 14.5–65.3)	3 months	NR	USD	2012
			Colorectal 52 (22–126)				
			Breast 49 (12–86)				
			Prostate 11 (3–19)				
Massa	2019	Total non-reimbursed cost of cancer patients	Median 929 (95% CI 775 to 1084) for HNC	1 year	Median 3.93% of total income spent on OOP (95% CI 3.21 to 4.65)	USD	2014
			918 (885 to 951) for other cancer				
Narang	2016	Costs paid by patients on inpatient hospitalization, nursing homes, clinic visits, outpatient surgery	3737 average	1 year	Uninsured: 23%	USD	2012
			2116 Medicaid		Medicaid: 8.5%		
			5492 employer-sponsored insurance		Employer-sponsored insurance: 12.6%		
			8115 uninsured		NR		
Newton	2018	Direct medical and nonmedical expenses borne by the patient	2179 (3077) (95% CI 1873–2518)	21 weeks	11% spent over 10% of household income	AUD	2016
O Ceilleachair	2017	OOPCs survivors had incurred as a result of their diagnosis, and which were not recouped from PHI or other sources	1589 (3827)	1 year	NR	Euro	2008
Olszewski	2017	Patient’s cost sharing on medication	No low-income subsidy: median 5623 (IQR 3882–9437)	1 year	23% of annual income among non-subsidized	USD	2012
			Low-income subsidy: median 6 (IQR 3–10)				
O-Neill	2015	Medical and nonmedical costs related to the hospital visit coinciding with the interview	717 (95% CI 619–1171)	1 year	>67% patients had catastrophic expenses (>40% of household income)	USD	2014
Pisu	2016	Out of pocket costs for medical care	Total at baseline: 232 (82)	1 month	NR	USD	2015
			Total at 3 months 186 (71)				
Pisu	2011	Expenses since diagnosis, including monthly insurance premiums	Total: 316.1 (411.5)	1 month	31% for lowest income level (<20,000 per year)	USD	2008
			Caucasian: 297 (296)				
			Minority: 204 (405)				
Raborn	2012	Deductibles and co-payments for anticancer medication	Generic versions: 171 (652)	Per claim	NR	USD	2009
			No available generic versions: 31 (130)				
Roberts	2015	Deductibles, copayments, and coinsurance payments	175 (484)	1 year	NR	USD	2012
Sculpher	2000	Travel expenses for treatment appointments	Treated with Raltitrexed: 12.25 (41.87) Treated with Fluorouracil: 10.70 (20.16)	Per patient-journey	NR	GBP	2000
Shen	2017	Patient out of pocket expenses on targeted oral anti-cancer medications	Median 401 (IQR 1029)	1 month	NR	USD	2014
Shih	2015	Patient OOP payments were calculated as allowed minus paid	USD 647 per month in 2011	1 month	NR	USD	2011
Shih	2017	Patient pay amount is the amount paid by beneficiaries that is not reimbursed by a third party; therefore, it captures the OOP payments for Medicare beneficiaries who are enrolled in the Part D program.	850	1 month	NR	USD	2012
Shiroiwa	2010	Co-payment	Patients JPY 328,000 (95% CI: 323,000–334,000)	11 months	NR	JPY	2009
			Patients ≥ 70 years JPY 61,000 (95% CI: 60,000–63,000)		NR		
Sneha	2017	Medical expenses and nonmedical out-of-pocket expenses incurred by the families in the course of care	NR	Per day	Non-medical expenses—Urban: 22%	INR	2012
					Rural: 46%		
Stommel	1992	Out-of-pocket payments for services: hospital and physician services, nursing homes, medications, visiting nurses, home health aides, and purchases of special equipment, supplies, and foods and supplements	660 (624)	3 months	NR	USD	1993
Suidan	2019	Patient out-of-pocket expenses, in addition to insurance payments made.	Neoadjuvant chemotherapy: USD 2519	8 months	NR	USD	2017
			Primary debulking: USD 2977		NR		
Tangka	2010	OOP cost (inpatient, outpatient, other noninpatient (costs related to emergency room visits, home healthcare, vision aids, and other medical supplies), Rx) attributable to cancer = difference between expenditures for persons with cancer and persons without cancer, adjusted for sociodemographic and comorbidities	3996	1 year	NR	USD	2007
Thompson	2019	Costs to items associated with the excision, including consultations, skin cancer treatment, Anatomical pathology, skin flaps and Anesthesia;Excluding bulk-billed patients were co-payment would be USD 0	Private clinical rms: 80 (34, 170)	Treatment episode (up to 3 days post-discharge)	NR	AUD	2018
			Public hospital: 35 (30, 104)				
			Private hospital: 350 (196, 596)				
Tomic	2013	Out-of-pocket costs for G-CSF per patient	100–150: pegfilgrastim 50–80: filgrastim	3 months	NR	USD	2010
Tsimicalis	2013	Direct costs included health services, prescription medications, over-the-counter medications, complementary medicines, supplies, equipment, family medical fees and medications, as well as travel, food, communication, accommodations, moving or renovations, provider for the child with cancer, domestic labour (e.g., sibling child care), funeral, and other cost categories not yet captured in the literature	730 (1520)	3 months	NR	CDN	2007
Tsimicalis	2012	Direct costs as well as travel, food, communication, accommodations, moving or renovations, provider for the child with cancer, domestic labour (e.g., sibling childcare), funeral, and other costs	5446 (6659)	3 months	NR	CDN	2007
Van Houtven	2010	Out-of-pocket expenditures for the patient’s medical care as well as nonmedical expenditures	Overall: 1243	By phase	NR	USD	2005
			Initial: 921		NR		
			Continuing: 1545		NR		
			Terminal: 1015		NR		
Wang	2014	Ward charges, laboratory charges, radiology charges, prescription charges, surgical charges, and other charges	2230 (95% CI: 1976–2483)	Per episode	NR	USD	2012
Wenhui	2017	NR	1878	NR	51.6	USD	2008
			1146	NR	NR		
			348	NR	NR		
Wood	2019	Direct out-of-pocket expenses were defined as wage losses (per week); non-medical expenses associated with general practitioner or hospital visits (in the last 3 months); costs of treatments for conditions linked to NSCLC (in the last week), such as those for pain or symptom relief; and other non-medical costs arising from the diagnosis (per week), including additional childcare costs, assistance at home (cleaner, housekeeper, gardener), and travel costs.	Patient: 823 Caregivers: 1019	3 months, reported as annual	NR	EUR	2018
Yu	2015	Out-of-pocket costs: costs paid by the patient/family for travel, supplies, medications, etc.	NR	Entire palliative trajectory	NR	CDN	2012

AUD = Australian dollar; CAD = Canadian dollar; CI = confidence interval; CNY = Chinese yuan; GBP = Great Britain Pound; INR = Indian Rupee; IQR = interquartile range; JPY = Japanese Yen; NR = not reported; OOP = out-of-pocket; RM = Renminbi; SD = standard deviation; USD = United States Dollar.

## Data Availability

Not applicable.

## Supplementary Materials

The following are available online at https://www.mdpi.com/1718-7729/28/2/117/s1, Supplementary 1: Search strategies, Supplementary 2: Total cost conversion and estimation, Supplementary 3: Cost conversion and estimation across categories, Supplementary 4: Preferred reporting items for systematic reviews and meta-analyses (PRISMA) checklist.
