# Supplementary material for: The Out-of-Pocket Cost Burden of Cancer Care—A Systematic Literature Review
Source: Curr Oncol. 2021 Mar 15;28(2):1216–48. doi: 10.3390/curroncol28020117 (PMC8025828; doi:10.3390/curroncol28020117)
Supplement: Supplementary file 1 [file curroncol-28-00117-s001.pdf]

Supplementary Material

Supplementary 1: Search strategies

1. **Medline**

Line	Search Term
1	Neoplasms/
2	neoplasm*.ti,ab.
3	cancer.ti,ab.
4	1 or 2 or 3
5	"Deductibles and Coinsurance"/
6	copay*.ti,ab.
7	co-pay*.ti,ab.
8	deductible*.ti,ab.
9	coinsurance.ti,ab.
10	co-insurance.ti,ab.
11	oop.ti,ab.
12	out-of-pocket.ti,ab.
13	out of pocket.ti,ab.
14	(pocket* adj2 cost*).ti,ab.
15	(transpo* adj2 cost*).ti,ab.
16	(travel* adj2 cost*).ti,ab.
17	(caregiv* adj2 cost*).ti,ab.
18	(care giv$ adj2 cost$).ti,ab.
19	health expenditures/ or capital expenditures/
20	5 or 6 or 7 or 8 or 9 or 10 or 11 or 12 or 13 or 14 or 15 or 16 or 17 or 18 or 19
21	4 and 20

<u>Total hits:</u> 1532

2. **Embase**

Line	Search terms
1	neoplasm/
2	neoplasm*.ti,ab.
3	cancer.ti,ab.
4	1 or 2 or 3
5	out-of-pocket.ti,ab.
6	out of pocket.ti,ab.
7	oop.ti,ab.
8	coinsurance.ti,ab.

Line	Search terms
9	co-insurance.ti,ab.
10	deductible*.ti,ab.
11	co-pay*.ti,ab.
12	copay*.ti,ab.
13	(pocket* adj2 cost*).ti,ab.
14	(transpo* adj2 cost*).ti,ab.
15	(travel* adj2 cost*).ti,ab.
16	(caregiv* adj2 cost*).ti,ab.
17	(care giv$ adj2 cost$).ti,ab.
18	5 or 6 or 7 or 8 or 9 or 10 or 11 or 12 or 13 or 14 or 15 or 16 or 17
19	4 and 18

Total hits: 1618

3. **EconLit**

Line	Search terms
1	Neoplasm
2	Cancer
3	1 OR 2
4	copay*
5	co-pay*
6	deductible*
7	coinsurance
8	oop
9	out-of-pocket
10	out of pocket
11	time cost
12	travel cost
13	transport* cost*
14	caregiv* cost*
15	4 OR 5 OR 6 OR 7 OR 8 OR 9 OR 10 OR 11 OR 12 OR 13 OR 14
16	3 AND 15

Total hits: 22

4. **CINAHL**

Line	Search terms
1	(MH "Neoplasms+"
2	"cancer*"

Line	Search terms
3	S2 OR S1
4	"copay*"
5	"co-pay*"
6	"deductible*"
7	"coinsurance"
8	"oop"
9	"out-of-pocket"
10	"out of pocket"
11	"time cost"
12	"travel cost"
13	"transport* cost*"
14	"caregiv* cost*"
15	S4 OR S5 OR S6 OR S7 OR S8 OR S9 OR S10 OR S11 OR S12 OR S13 OR S14

<u>Total hits:</u> 467

Supplementary 2: Total cost conversion and estimation

First Author	Country	Sample size	Outcome (USD 2018)	SD (USD 2018)	95% CI lower (USD 2018)	95% CI upper (USD 2018)	Weight	Weighted OOP
O Ceilleachair	Ireland	497	168	404	132	203	0.00156	0.26
Ahuja	India	11	201	110	136	266	0.00003	0.01
Chin	USA	1025	650	267	634	667	0.00321	2.09
Baili	Italy	296	183	425	134	231	0.00093	0.17
Bates	Australia	25530	58	116	56	59	0.07994	4.62
Bekelman	USA	15643	308	1510	284	332	0.04898	15.08
Bercow	USA	5031	269	229	263	275	0.01575	4.24
Buttner	Germany	502	76	128	65	87	0.00157	0.12
Callander	Australia	429	69	179	52	86	0.00134	0.09
Shen	USA	898	425	808	372	478	0.00281	1.20
Chang	USA	58	402	843	185	619	0.00018	0.07
Cohn	Australia	100	480	807	322	638	0.00031	0.15
Davidoff	USA	1868	238	10	238	239	0.00585	1.39
de Oliveira	Canada	585	15	86	8	22	0.00183	0.03
Dusetzina	USA	1541	121	337	104	138	0.00483	0.58
Farias	USA	6863	193	97	191	195	0.02149	4.15
Finkelstein	USA	679	186	291	164	208	0.00213	0.40
Ghatak	India	50	566	306	481	651	0.00016	0.09
Gordon	Australia	287	139	230	112	165	0.00090	0.12
Gordon	Australia	539	12	11	11	13	0.00169	0.02
Gordon	Australia	419	103	126	90	115	0.00131	0.13
Gordon	Australia	439	258	313	229	288	0.00137	0.36
Gordon	Australia	289	438	693	358	518	0.00090	0.40

First Author	Country	Sample size	Outcome (USD 2018)	SD (USD 2018)	95% CI lower (USD 2018)	95% CI upper (USD 2018)	Weight	Weighted OOP
Grange	France, Germany and the United Kingdom	186	380	841	259	500	0.00058	0.22
Grange	France, Germany and the United Kingdom	186	609	772	498	720	0.00058	0.35
Gupta	USA	162	236	436	169	303	0.00051	0.12
Guy	USA	4271	197	609	179	215	0.01337	2.64
Hanly	Ireland	154	404	764	283	525	0.00048	0.19
Hess	USA	47207	334	24558	112	555	0.14781	49.35
Housser	Canada	151	264	297	216	311	0.00047	0.12
Housser	Canada	151	251	354	194	307	0.00047	0.12
Kumthekar	USA	43	2598	2672	1799	3397	0.00013	0.35
Jayadevappa	USA	512	269	958	186	352	0.00160	0.43
Jung	USA	148265	347	153	347	348	0.46423	161.27
Kaisaeng	USA	3781	180	476	165	195	0.01184	2.13
Langa	USA	988	640	535	607	674	0.00309	1.98
Lansky	USA	70	775	747	600	950	0.00022	0.17
Lauzier	Canada	1191	112	101	106	118	0.00373	0.42
Leopold	USA	2682	386	988	348	423	0.00840	3.24
Leopold	USA	2682	603	1269	555	651	0.00840	5.07
Longo	Canada	141	400	847	260	540	0.00044	0.18
Longo	Canada	141	152	270	107	197	0.00044	0.07
Mahal	India	821	16	36	14	19	0.00257	0.04
Marti	UK	75	14	81	0	33	0.00023	0.00
Marti	UK	75	19	96	0	41	0.00023	0.00
Marti	UK	75	18	118	0	45	0.00023	0.00

First Author	Country	Sample size	Outcome (USD 2018)	SD (USD 2018)	95% CI lower (USD 2018)	95% CI upper (USD 2018)	Weight	Weighted OOP
Marti	UK	75	4	26	0	10	0.00023	0.00
Massa	USA	489	82	153	68	96	0.00153	0.13
Massa	USA	16771	81	193	78	84	0.05251	4.26
Newton	Australia	400	305	431	263	347	0.00125	0.38
Olszewski	USA	1519	511	374	492	530	0.00476	2.43
Olszewski	USA	1519	1	0	1	1	0.00476	0.00
O-Neill	Haiti	61	63	34	55	72	0.00019	0.01
Pisu	USA	216	246	87	234	258	0.00068	0.17
Pisu	USA	216	197	75	187	207	0.00068	0.13
Pisu	USA	87	370	481	269	471	0.00027	0.10
Pisu	USA	87	347	346	275	420	0.00027	0.09
Pisu	USA	87	239	474	139	338	0.00027	0.07
Roberts	USA	18575	16	44	15	17	0.05816	0.92
Shiroiwa	Japan	659	289	82	282	295	0.00206	0.60
Shiroiwa	Japan	659	54	179	40	67	0.00206	0.11
Stommel	USA	192	383	362	332	434	0.00060	0.23
Tsimicalis	Canada	78	219	456	118	320	0.00024	0.05
Tsimicalis	Canada	99	1634	1998	1240	2027	0.00031	0.51

Supplementary 3: Cost conversion and estimation across categories

			Medications costs (in 2018 USD)			
First Author	ID	Sample size	Mean/month	SD	95% confidence interval lower limit	95% confidence interval upper limit
O Ceilleachair	2	497	51	96		
Ahuja	3	11	48			
Azzani	6	138	111			
Baili	7	296	40	76		
Bao	8	3825	1254			
Bercow	13	5031	32	13		
Buttner	18	502	9			
Calhoun	19	83	212			
Callander	20	429	34	38		
Shen	21	898	425	312		
Cohn	26	100	18			
Darkow	29	995	71			
Dumont	35	252	23			
Dusetzina	36	63780	156			
Dusetzina	37	3344	329			
Farias	40	6863	17	22		
Geynisman	42	116	81			
Ghatak	43	50	415	93		
Giordano	44	14643	194			
Gordon	48	419	12			
Gordon	49	439	44	156		
Gupta	53	162	176	238		
Hanly	55	154	57	152		
Housser	57	151	42	68		
		151	154	273		
Kumthekar	61	43	753	2213		
Jayadevappa	64	512	43	69		
Jung	65	148265	347	153		
John	66	2977	25			

			Medications costs (in 2018 USD)			
First Author	ID	Sample size	Mean/month	SD	95% confidence interval lower limit	95% confidence interval upper limit
Kaisaeng	67	3781	180	476		
Kodama	69	577	1126			
Koskinen	70	1978	23			
Langa	71	988	193	546		
Lauzier	73	1191	29	41		
Longo	76	141	58	187		
		141	41	121		
Marti	79	75	4	3		
Newton	82	400	20	32		
Olszewski	83	1519	511	374		
		1519	1	0		
O-Neill	84	61	23	21	18	47
Pisu	86	87	54	82		
		87	55	89		
		87	48	34		
Roberts	88	18575	16	44		
Shiroiwa	91	659	289	82	284	294
		659	54	179	44	55
Tangka	95	24654	112			
Tomic	97	28979	48			
Tsimicalis	99	99	22	46		
Shih	104	42111	927			

			Clinical consultations (in 2018 USD)			
First Author	ID	Sample size	Mean/month	SD	95% confidence interval lower limit	95% confidence interval upper limit
O-Ceilleachair	2	497	76	43		
Baili	3	11	11	21		
Calhoun	6	138	109			

			Clinical consultations (in 2018 USD)			
First Author	ID	Sample size	Mean/month	SD	95% confidence interval lower limit	95% confidence interval upper limit
Callander	7	296	21	19		
Cohn	8	3825	22			
Gordon	13	5031	16			
Gordon	18	502	44	55		
Gupta	19	83	6	212		
Langa	20	429	59	97		
Lauzier	21	898	31	53		
Longo	26	100	82	601		
Newton	29	995	27	36		
Pisu	35	252	113	168		
Pisu	36	63780	102	110		
Pisu	37	3344	163	316		
Tangka	40	6863	213			
Tsimicalis	42	116	5	22		

			Medical procedures/Sugery (in 2008 USD)			
First Author	ID	Sample size	Mean/month	SD	95% confidence interval lower limit	95% confidence interval upper limit
O Ceilleachair	2	497	65	48		
Azzani	6	138	131			
Baili	7	296	5	27		
Bekelman	12	15643	56	14		
Bercow	13	5031	53	114		
Boyages	16	361	59			
Buttner	18	502	35			
Calhoun	19	83	1149			

			Medical procedures/Sugery (in 2008 USD)			
First Author	ID	Sample size	Mean/month	SD	95% confidence interval lower limit	95% confidence interval upper limit
Callander	20	429	5	29		
Ghatak	43	50	120	286		
Gordon	46	287	95	194	73	120
Gordon	47	539	12	11		
Gordon	48	419	60			
Gordon	50	289	261	244		
Hanly	55	154	201	592		
Kumthekar	61	43	427	881		
John	66	2977	32			
Langa	71	988	138	247		
Lauzier	73	1191	59	83		
Narang	81	352	23			
Narang	81	352	82			
Narang	81	352	273			
Newton	82	400	109	276		
O-Neill	84	61	3	5	2	12
Tangka	95	24654	19			

			Transportation/travel cost estimate (in 2018 USD)			
First Author	ID	Sample size	Mean/month	SD	95% confidence interval lower limit	95% confidence interval upper limit
Ahuja	3	11	18			
Azzani	6	138	11			
Baili	7	296	71	349		
Buttner	18	502	29			
Cohn	26	100	33			
Collins	28	151	65			
Dumont	35	252	20			

			Transportation/travel cost estimate (in 2018 USD)			
First Author	ID	Sample size	Mean/month	SD	95% confidence interval lower limit	95% confidence interval upper limit
Gordon	49	439	184	262		
Grange	51	186	0	0	0	0
Grange	51	186	380	841	310	458
Grange	51	186	609	772	545	679
Gupta	53	162	22	38		
Housser	57	151	156	179		
Housse	57	151	152	194		
Houts	58	139	7			
Isshiki	60	521	84			
Kumthekar	61	43	347			
Koskinen	70	1978	3			
Lansky	72	70	245	275		
Marti	79	75	3	2		
Newton	82	400	64	90		
O-Neill	84	61	11	6	9	20
Pisu	86	87	51	68		
Pisu	86	87	38	69		
Pisu	86	87	46	59		
Sneha	92	70	230			
Sneha	92	70	801			
Tsimicalis	98	78	150	312		
Tsimicalis	99	99	920	1746		

			Caregiver cost estimate (in 2018 USD)			
First Author	ID	Sample size	Mean/month	SD	95% confidence interval lower limit	95% confidence interval upper limit
Ahuja	3	11	24			
Azzani	6	138	19			
Baili	7	296	48	189		
Chang	22	58	402	843		

			Caregiver cost estimate (in 2018 USD)			
First Author	ID	Sample size	Mean/month	SD	95% confidence interval lower limit	95% confidence interval upper limit
Cohn	26	100	367			
Collins	28	151	101			
Gordon	46	287	24	77	15	32
Gordon	49	439	68	79		
Lansky	72	70	27	73		
Lauzier	73	1191	28	43		
Longo	76	141	39	139		
Longo	76	141	3	30		
Newton	82	400	23	26		
Newton	82	87	87	19		
Sneha	92	70	93			
Sneha	92	70	62			
Tsimicalis	99	99	3412	1499		
Tsimicalis	99	99	1496	1398		

Supplementary 4: Preferred reporting items for systematic reviews and meta-analyses (PRISMA) checklist

Title	1	Identify the report as a systematic review, meta-analysis, or both.	1
Structured summary	2	Provide a structured summary including, as applicable: background; objectives; data sources; study eligibility criteria, participants, and interventions; study appraisal and synthesis methods; results; limitations; conclusions and implications of key findings; systematic review registration number.	2
Rationale	3	Describe the rationale for the review in the context of what is already known.	3-4
Objectives	4	Provide an explicit statement of questions being addressed with reference to participants, interventions, comparisons, outcomes, and study design (PICOS).	3-4
Protocol and registration	5	Indicate if a review protocol exists, if and where it can be accessed (e.g., Web address), and, if available, provide registration information including registration number.	5
Eligibility criteria	6	Specify study characteristics (e.g., PICOS, length of follow-up) and report characteristics (e.g., years considered, language, publication status) used as criteria for eligibility, giving rationale.	5
Information sources	7	Describe all information sources (e.g., databases with dates of coverage, contact with study authors to identify additional studies) in the search and date last searched.	6
Search	8	Present full electronic search strategy for at least one database, including any limits used, such that it could be repeated.	49-51
Study selection	9	State the process for selecting studies (i.e., screening, eligibility, included in systematic review, and, if applicable, included in the meta-analysis).	5
Data collection process	10	Describe method of data extraction from reports (e.g., piloted forms, independently, in duplicate) and any processes for obtaining and confirming data from investigators.	6
Data items	11	List and define all variables for which data were sought (e.g., PICOS, funding sources) and any assumptions and simplifications made.	7
Risk of bias in individual studies	12	Describe methods used for assessing risk of bias of individual studies (including specification of whether this was done at the study or outcome level), and how this information is to be used in any data synthesis.	7
Summary measures	13	State the principal summary measures (e.g., risk ratio, difference in means).	6-7
Synthesis of results	14	Describe the methods of handling data and combining results of studies, if done, including measures of consistency (e.g., I^2) for each meta-analysis.	6-7

Risk of bias across studies	15	Specify any assessment of risk of bias that may affect the cumulative evidence (e.g., publication bias, selective reporting within studies).	7
Additional analyses	16	Describe methods of additional analyses (e.g., sensitivity or subgroup analyses, meta-regression), if done, indicating which were pre-specified.	NA

Study selection	17	Give numbers of studies screened, assessed for eligibility, and included in the review, with reasons for exclusions at each stage, ideally with a flow diagram.	8
Study characteristics	18	For each study, present characteristics for which data were extracted (e.g., study size, PICOS, follow-up period) and provide the citations.	8
Risk of bias within studies	19	Present data on risk of bias of each study and, if available, any outcome level assessment (see item 12).	11
Results of individual studies	20	For all outcomes considered (benefits or harms), present, for each study: (a) simple summary data for each intervention group (b) effect estimates and confidence intervals, ideally with a forest plot.	18-34
Synthesis of results	21	Present results of each meta-analysis done, including confidence intervals and measures of consistency.	9-10
Risk of bias across studies	22	Present results of any assessment of risk of bias across studies (see Item 15).	39
Additional analysis	23	Give results of additional analyses, if done (e.g., sensitivity or subgroup analyses, meta-regression [see Item 16]).	NA
Summary of evidence	24	Summarize the main findings including the strength of evidence for each main outcome; consider their relevance to key groups (e.g., healthcare providers, users, and policy makers).	12-14
Limitations	25	Discuss limitations at study and outcome level (e.g., risk of bias), and at review-level (e.g., incomplete retrieval of identified research, reporting bias).	15
Conclusions	26	Provide a general interpretation of the results in the context of other evidence, and implications for future research.	16
Funding	27	Describe sources of funding for the systematic review and other support (e.g., supply of data); role of funders for the systematic review.	1
